# CHARACTERIZING PERFORMANCE VARIABILITY DURING A SIX-MINUTE WALK TEST

**DOI:** 10.1093/geroni/igae098.3464

**Published:** 2024-12-31

**Authors:** Tina Brinkley, Fang-Chi Hsu, Dalane Kitzman, Denise Houston, Jason Fanning

**Affiliations:** Wake Forest University School of Medicine, Winston-Salem, North Carolina, United States; Wake Forest University School of Medicine, Winston-Salem, North Carolina, United States; Wake Forest University School of Medicine, Winston-Salem, North Carolina, United States; Wake Forest University School of Medicine, Winston Salem, North Carolina, United States; Wake Forest University, Winston-Salem, North Carolina, United States

## Abstract

The six minute walk (6MW) is a test of sub-maximal exercise capacity that reflects the level at which most activities of daily living are performed. While 6MW outcomes traditionally focus on distance covered or average gait speed, recent efforts explore methods to capture performance variability. We used functional principal component analysis (fPCA) to characterize patterns of lap-to-lap performance in 33 adults with overweight/obesity (mean age: 64.2±7.7 yrs, mean BMI: 35.5±6.1 kg/m2, 79% male, 82% White). The 6MW was performed on a 20-m course using standardized procedures, and the time to complete each half lap was recorded. Spearman correlation coefficients were used to examine associations between fPCA components and physical function measures including the expanded Short Physical Performance Battery (eSPPB) and self-reported mobility. Two fPCA components were extracted, explaining 72% (PC1) and 15% (PC2) of the variance in lap-to-lap performance. PC1 captured both speed and consistency of performance, where higher scores were correlated with greater 6MW distance (r=0.93, p< 0.0001), faster initial 6MW gait speed (r=0.80, p< 0.0001), 4-meter usual gait speed (r=0.64, p< 0.0001), and repeated chair stands time (r=-0.40, p=0.02), and higher eSPPB (r=0.50, p-0.027) and mobility (r=0.54, r=0.001) scores. PC2 captured consistency in performance, with higher scores correlating with less gait change speed during the 6MW (r=-0.63, p< 0.0001) and better balance (r=0.43, p=0.0122). These results suggest faster gait speed overall as well as during the initial stages of the 6MW are associated with better physical function. Future research should examine whether intervening on gait speed and gait consistency benefits long-term independence.

